# Optimization of the Linker Length of Mannose-Cholesterol Conjugates for Enhanced mRNA Delivery to Dendritic Cells by Liposomes

**DOI:** 10.3389/fphar.2018.00980

**Published:** 2018-09-05

**Authors:** Fazhan Wang, Wen Xiao, Mostafa A. Elbahnasawy, Xingting Bao, Qian Zheng, Linhui Gong, Yang Zhou, Shuping Yang, Aiping Fang, Mohamed M. S. Farag, Jinhui Wu, Xiangrong Song

**Affiliations:** ^1^State Key Laboratory of Biotherapy, Geriatrics and Cancer Center, West China Hospital, and Collaborative Innovation Center for Biotherapy, Sichuan University, Chengdu, China; ^2^Department of Botany and Microbiology, Faculty of Science, Al-Azhar University, Cairo, Egypt

**Keywords:** mRNA vaccine, dendritic cell targeting liposomes, mannose conjugates, linker length, click reaction

## Abstract

Liposomes (LPs) as commonly used mRNA delivery systems remain to be rationally designed and optimized to ameliorate the antigen expression of mRNA vaccine in dendritic cells (DCs). In this study, we synthesized mannose-cholesterol conjugates (MP_n_-CHs) by click reaction using different PEG units (PEG_100_, PEG_1000_, and PEG_2000_) as linker molecules. MP_n_-CHs were fully characterized and subsequently used to prepare DC-targeting liposomes (MP_n_-LPs) by a thin-film dispersion method. MP_n_-LPs loaded with mRNA (MP_n_-LPX) were finally prepared by a simple self-assembly method. MP_n_-LPX displayed bigger diameter (about 135 nm) and lower zeta potential (about 40 mV) compared to MP_n_-LPs. The *in vitro* transfection experiment on DC2.4 cells demonstrated that the PEG length of mannose derivatives had significant effect on the expression of GFP-encoding mRNA. MP_1000_-LPX containing MP_1000_-CH can achieve the highest transfection efficiency (52.09 ± 4.85%), which was significantly superior to the commercial transfection reagent Lipo 3K (11.47 ± 2.31%). The optimal DC-targeting MP_1000_-LPX showed an average size of 132.93 ± 4.93 nm and zeta potential of 37.93 ± 2.95 mV with nearly spherical shape. Moreover, MP_1000_-LPX can protect mRNA against degradation in serum with high efficacy. The uptake study indicated that MP_1000_-LPX enhanced mRNA expression mainly through the over-expressing mannose receptor (CD206) on the surface of DCs. In conclusion, mannose modified LPs might be a potential DC-targeting delivery system for mRNA vaccine after rational design and deserve further study on the *in vivo* delivery profile and anti-tumor efficacy.

## Introduction

Messenger RNA (mRNA) has recently generated great attention as one of promising therapeutics with the potential for cancer immunotherapy and vaccines because the *in vitro*-transcribed (IVT) mRNA does not need to enter the nucleus and induces only transient protein expression without the risk of genomic integration compared with the widely investigated DNA (Sahin et al., 2014; Pardi et al., 2018). Some mRNA vaccines have been demonstrated to be effective in the preclinical mouse models of cancer (Kreiter et al., 2015; Oberli et al., 2017; Sayour et al., 2017). Nevertheless, the anionic character of mRNA does not facilitate its penetration into cells, resulting in low antigen expression and curative effect. It has been shown that the cellular uptake rate of naked mRNA is less than 1 in 10,000 molecules (Sahin et al., 2014). Moreover, mRNA is prone to degradation by RNases present everywhere (Tsui et al., 2002). Thus, sufficiently efficacious delivery system is urgently required to target antigen presentation cells (APCs) and protect mRNA from nuclease degradation, which will be beneficial for the clinical application of more mRNA vaccines (Pardi et al., 2018).

Non-viral vectors such as lipids, lipid-like materials, polymer or hybrid systems are widely studied for delivery of mRNA vaccines, which have low unwanted immune responses in contrast to the viral systems including adeno- associated viruses, lentiviruses and the Sendai virus (Giacca and Zacchigna, 2012; Midoux and Pichon, 2015; Hajj and Whitehead, 2017). Liposomes (LPs) are the most appealing and commonly used non-viral carriers of mRNA vaccines (Markov et al., 2015; Kranz et al., 2016; Persano et al., 2017; Verbeke et al., 2017). The mRNA loaded LPs namely RNA-LPX for cancer immunotherapy have been in phase I dose-escalation trial (Kranz et al., 2016). RNA-LPX protected mRNA from RNases and the encoded antigen can be efficiently expressed in the specialized APCs, like DCs (Kranz et al., 2016). Furthermore, the antigen-specific T-cell responses were also induced in melanoma patients. However, only 1 in 3 patients showed regression of a suspected metastatic thoracic lymph node lesion. The limited antitumor efficacy of RNA-LPX indicated that the LPs were worthy of being further reformed by functionalization of particles with ligands targeting DCs.

Dendritic cells express several mannose residue-recognizing membrane lectins like CD206 (mannose receptor, MR), CD209 (DC-SIGN) and CD207 (langerin) (Caminschi et al., 2012; Le Moignic et al., 2018). Macrophages as also expressed CD206 receptor (Chen et al., 2016; Kim et al., 2017) with the ability to present antigens (Malissen et al., 2014). They can mediate endocytosis of cargos encapsulated in mannose-modified nano-preparations (Li et al., 2013; Chen et al., 2014; Wang C. et al., 2014). Of note, enhanced *in vivo* anticancer efficacy via mannose modification on the nano-preparations has been widely reported in the literatures (Lai et al., 2018; Le Moignic et al., 2018; Yang et al., 2018). LPs can be easily modified because phospholipids and cholesterol are typically included (Hua and Wu, 2013). These lipophilic molecules can conjugate with various moieties binding to surface receptors of the target cells with high selectivity. Our previous studies demonstrated that folic acid-conjugating LPs can specifically deliver DNA into folate receptor-overexpressing tumor (He Z.Y. et al., 2013; Yang et al., 2016). The targeting molecule folic acid was linked to cholesterol, which efficiently kept its binding specificity to folate receptor (He Z. et al., 2013). Taking into account of these, mannosylated cholesterol derivatives were designed and synthesized to prepare mannosylated LPs to help delivery to DCs in this study.

According to literatures, the length and flexibility of the space between ligand molecules and the surface of particles might be important parameters for efficient recognition of receptors (Engel et al., 2003; Stefanick et al., 2013; Jeong et al., 2014). A short linker may restrict the translational freedom of ligand, while the longer one might bury a large fraction of the conjugated ligand (Stefanick et al., 2013). The optimal linker provides a more effective ligand-receptor interaction (Stefanick et al., 2013; Jeong et al., 2014). Thus, a rational design of the targeting LPs is crucial to enhanced mRNA delivery to DCs.

In our study, MP_n_-CHs containing different PEG units were firstly synthesized and then used to prepare the MP_n_-LPs by a typical thin-film dispersion method. The mRNA encapsulating liposomes (MP_n_-LPX) were constructed by complexing the obtained MP_n_-LPs and mRNA. The preferable MP_n_-LPX were picked out according to the *in vitro* transfection efficiency of GFP-encoding mRNA on DCs. The pharmaceutical properties and preliminary cytotoxicity of the optimal delivery system were also assessed to favor its potential application for mRNA delivery.

## Materials and Methods

### Materials

1,2,3,4,6-Penta-*O*-acetyl-alpha-D-mannopyranoside was obtained from Jinan Samuel Pharmaceutical Co., Ltd. (Shandong, China). Cholesterol was supplied from Shanghai Yuanju Biology Technology Company (Shanghai, China). Cholesterol-PEG_2000_-N_3_ was purchased by Shanghai Ponsure Biotech, Inc (Shanghai, China). 1,2-dioleoyl-3-trimethylammonium-propane (DOTAP) and 1,2-dioleoyl-sn-glycero-3-phosphoethanolamine (DOPE) was provided by Shanghai A.V.T. Pharmaceutical Co., Ltd. (Shanghai, China). GFP-mRNA was obtained from TriLink (San Diego, CA, United States). DMEM and fetal bovine serum (FBS) were purchased from Gibco. All the other chemical reagents were of analytical grade or better without further purification unless otherwise stated.

### Cell Culture

DC2.4 cells were cultured in DMEM medium supplemented with 10% FBS and 1% penicillin/streptomycin with 5% CO_2_ at 37°C in a humidified atmosphere.

### Synthesis and Characterization of MP_n_-CH

#### Synthesis of MP_100_-CH

**FIGURE 1:**
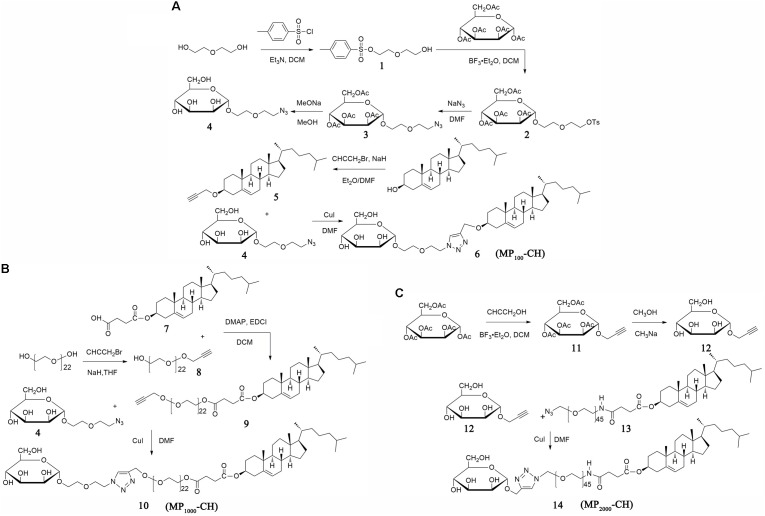
Synthesis of MP_n_-CHs. Schematic of the synthetic steps for MP_100_-CH **(A)**, MP_1000_-CH **(B)**, and MP_2000_-CH **(C)**, respectively.

As shown in **Figure 1A**, MP_100_-CH was obtained with similar procedures as described previously (Kim et al., 2012; Nguyen et al., 2016). In brief, Diethylene glycol (5.0 eq), paratoluensulfonyl chloride (TosCl, 1.0 eq) and triethylamine (TEA, 1.1 eq) were dissolved in anhydrous dichloromethane (DCM) and stirred for 24 hours (h) at room temperature (rt). The crude product was purified by silica gel with a mixed solvent system of DCM and methanol to harvest compound **1**. 1,2,3,4,6-Penta-*O*-acetyl-alpha-D-mannopyranoside (1.5 eq), compound 1 (1.0 eq) and BF_3_⋅Et_2_O (1.5 eq) were dissolved in anhydrous DCM. Compound **2** was acquired and purified by column chromatography. Compound 2 (1.0 eq) and sodium azide (5.0 eq) were added into anhydrous N, *N*-dimethyl-Formamide (DMF) and stirred suitably for 24 h at 60°C to prepare compound **3**. Subsequently, deacetylation of compound 3 was performed in methanol solution (HPLC grade) of sodium methoxide (NaOMe) (10:1, v/v). Then the reaction system was treated with (H+) resin to get product named compound **4**. Compound **5** was prepared as described previously (Rull-Barrull et al., 2016). In brief, Cholesterol (1.0 eq) and 3-bromopropyne (2.0 eq) were dissolved in component solvent containing anhydrous ether and anhydrous DMF (1:1, v/v). Sodium hydride (NaH, 5.0 eq) was added mildly and the solution was stirred at rt for 24 h. Finally, compound 4, compound 5 and copper iodide were mixed in equal molar ratio and dissolved in anhydrous DMF. The mixture was reacted for 24 h at rt and concentrated to obtained compound **6** (MP_100_-CH) via column chromatography.

#### Synthesis of MP_1000_-CH

The synthetic scheme was shown in **Figure 1B** and specific experimental steps were as follows. Compound **7** was prepared and purified according to our previously reported method (He Z.Y. et al., 2010). PEG_1000_ (5.0 eq), propargyl bromide (2.0 eq) and hydrogenated sodium (NaH, 3.0 eq) were dissolved in anhydrous tetrahydrofuran (THF). The mixture was stirred at rt overnight. Compound **8** was purified by column chromatography. Compound 7 (1.2 eq), 8 (1.0 eq), DMAP (0.5 eq), and EDCI (2.0 eq) were dissolved in DCM and stirred at rt for 24 h. Compound **9** was obtained after the crude product was purified by column chromatography. Similar to the synthetic method of compound 6, compound 4 was connected to compound 9, and compound **10** (MP_1000_-CH) was obtained.

#### Synthesis of MP_2000_-CH

Compound 14 were prepared according to the scheme showed in **Figure 1C**. Briefly, compound **11** was prepared according to the synthesis method of compound 2 but replace the compound 1 with propargyl alcohol. Subsequently, compound **12** was acquired using the similar synthesis method of compound 4. Compound **14** (MP_2000_-CH) was obtained with the similar synthesis method of compound 10 after column chromatography.

#### General Characterization of Prepared Compounds

^1^H-NMR spectra of MP_n_-CH and other prepared compounds dissolved in CDCl_3_, D_2_O or Dimethyl Sulfoxide-D_6_ containing TMS were recorded on a Unity Inova-400 (400 MHz) (Varian Inc., Palo Alto, CA, United States). Chemical shifts were analyzed in ppm relative to the residual solvent peaks of TMS. The mass spectra of various compounds were obtained using a Waters Q-TOF Premier (Milford, MA, United States) equipped with the ion spray source and using N_2_ as nebulization gas. In addition, the identity of the conjugate was also verified by Fourier Transform infrared spectroscopy (FTIR) using a Vector 22 spectrometer (Bruker, Ettlingen, Switzerland). The purity and retention time of MP_n_-CHs and other cholesterol derivatives were evaluated by high performance liquid chromatography (HPLC, Waters, Milford, MA, United States) at 201 nm. The mobile phase, at 1 mL/min flow rate, was composed of 100% chromatographic methanol. The retention time and purity of cholesterol derivatives were summarized in **Table 1**.

**Table 1:** The retention time and purity of cholesterol derivatives was evaluated by HPLC.

	Peak 1		Peak 2		Peak 3	
	Time (min)	Purity (%)	Time (min)	Purity (%)	Time (min)	Purity (%)
Compound **6**	**9.743**	**97.948**	10.493	1.996	–	–
Compound **9**	7.14	2.9	11.557	2.204	**16.692**	**94.881**
Compound **10**	7.548	1.627	11.556	1.878	**13.122**	**96.495**
Compound **13**	–	–	10.278	8.749	13.662	91.251
Compound **14**	**6.907**	**91.532**	10.896	2.321	13.796	6.147

*Bold values represents the retention time and purity of each compound*.

### Preparation and Characterization of MP_n_-LPX

#### Preparation of MP_n_-LPs

The cationic LPs were prepared using a thin-film dispersion method with some modifications (Wang F. et al., 2018). Briefly, cationic lipid DOTAP, the helper lipid DOPE, CH and MP_n_-CHs at a molar ratio of 50:10:35:5 or 50:10:40:0 (**Table 2**) were dissolved in a mixture solvent of chloroform/ethanol (1:1, v/v) to prepare MP_n_-LPs and LPs, respectively. The organic solvents were evaporated using a rotary evaporator at 37°C for 2 h. The lipid film was rehydrated with 2 mL RNase-free water at 60°C for 40 min to obtain a suspension with the final lipid concentration of 6 mM. Subsequently, the above suspension was sonicated at 80 W for 3 min and filtered with a 0.22 μm sterilized filter for the following experiment. The coumarin-6 (Cou-6) loaded LPs were acquired using the similar procedure with the addition of Cou-6 into the chloroform/ethanol (1:1 v/v) solvent mixture. The fluorescent intensity of Cou-6 loaded particles was measured using CyrationTM3 (BioTek Instruments, Inc, United States).

**Table 2:** Formulation component and relative molar content used in the manuscript.

	LPs	MP_100_-LPs	MP_1000_-LPs	MP_2000_-LPs
DOTAP	50	50	50	50
DOPE	10	10	10	10
CH	40	35	35	35
MP_100_-CH	0	5	0	0
MP_1000_-CH	0	0	5	0
MP_2000_-CH	0	0	0	5

#### Preparation of MP_n_-LPX

In our study, MP_n_-LPX was composed of DOTAP contained LPs and mRNA at N/P ratio of 3, 5 or 7, named MP_n_-LPX NP 3, MP_n_-LPX NP 5 or MP_n_-LPX NP 7, respectively. MP_n_-LPX were prepared according to previous reported methods with some modifications (Kranz et al., 2016). Briefly, mRNA was diluted by water and 1.5 M NaCl followed by adding corresponding MP_n_-LPs diluted with water to reach the desired ratio of N/P with the final concentration of NaCl of 150 mM. After incubated at rt for 15 min, MP_n_-LPX were finally obtained.

#### Size and Zeta Potential Measurements

The average particle size, size distribution (polydispersity index, PDI) and zeta potential of different formulations were recorded by Zetasizer Nano ZS90 (Malvern Instruments, Malvern, United Kingdom). All measurements were carried out using diluted samples at 25°C and were conducted in triplicate.

### Cellular Transfection of MP_n_-LPX

To optimize the appropriate ratio of N/P, DC2.4 cells in the logarithmic growth period were seeded in 24 well plates at 4 × 10^5^ cells/well and incubated for 24 h at 37°C, followed by incubation with different N/P of LPX (0.5 μg GFP-mRNA per well) in triplicate from 3 to 7. Before transfection, the culture medium was replaced with 500 μL FBS-free DMEM. Subsequently, LPX were added. After 4 h of incubation, 500 μL complete medium was added, and the cells were incubated for another 20 h. Expression of GFP by DC2.4 cells was visualized using a fluorescence microscope (Olympus Corp., Tokyo, Japan) and transfection efficiency was obtained based on the percentage of GFP positive cells from the live cell population by flow cytometry. Additionally, mean GFP fluorescence intensity of individual cells from GFP positive cells population after transfection was measured using FlowJo software (Li et al., 2017b).

To further investigate the transfection efficiency of MP_n_-LPX, DC2.4 cells in 24 well plates were incubated with MP_n_-LPX with the N/P of 5 following the same procedure described above. Transfection efficiency and mean fluorescence intensity (MFI) of GFP positive DC2.4 cells were evaluated by flow cytometry. In brief, DC2.4 cells were captured via forward scatter (FSC) and side scatter (SSC). Live DC2.4 cells were gated as shown in Region 1 (R1), of which GFP positive cells were selected (R2). Transfection efficiency (% GFP^+^ cells) was auto displayed with R2. MFI of GFP expression in GFP positive cells was acquired using FlowJo software. MFI was calculated after subtraction of background values of untreated DC2.4 cells. To further elaborate the kinetics of mRNA transfection *in vitro*, transfection efficiency of MP_1000_-LPX NP 5 on DC2.4 cells from 12 to 72 h has also been studied.

### Characterization of Optimal MP_1000_-LPX

#### Microscopy Investigation

The appearance and Tyndall effect of MP_1000_-LX were recorded by a digital camera. The morphology of MP_1000_-LPX NP 5 was examined by transmission electron microscopy (TEM, H-600, Hitachi, Japan). Briefly, 100 μL of MP_1000_-LPX suspension was added onto copper electron microscopy grids. Subsequently, they were negatively stained with 2% phosphotungstic acid for observation.

#### Gel Electrophoresis Retardation Assay

To evaluate the complexation of mRNA and MP_1000_-LPs, 1 μg free mRNA and MP_1000_-LPX (containing 1 μg mRNA) were diluted with RNase-free water. Then NorthernMax^®^ formaldehyde load dye containing ethidium bromide (50 μg/mL) was added and mixed. After incubating the samples for 10 min in 65°C, the samples were loaded into a 1% denaturing formaldehyde agarose gel in precooled MOPS buffer. The gel was run for 20 min at 180 V and analyzed using a molecular imager, ChemiDocTM 219 XRS system (Bio-Rad, United States). RNA Millennium^TM^ markers (Ambion) with bands at a range of 0.5–9 kb was included to provide size determination of the mRNA.

#### Stability Assay

For storage stability experiments, prepared MP_n_-LPX NP 5 were stored at 4°C for 1 and 3 days and another 1 h at rt before particle size and transfection efficiency measurement following the similar procedure described previously (Kranz et al., 2016).

To evaluate the serum stability, 1 μg free mRNA and MP_n_-LPX NP 5 (containing 1 μg mRNA) were incubated in parallel with 150 mM NaCl supplemented with or without FBS at 50% final concentration at 37°C for 2 h, respectively. To release mRNA from LPX, 1 μL of 10% Triton X-100 was added to 10 μL of MP_1000_-LPX samples and incubated at rt for 10 min. After mixed with NorthernMax^®^ formaldehyde load dye, samples were treated and visualized using the similar process as described in Section “Gel Electrophoresis Retardation Assay.”

#### Cytotoxicity Assay

To test potential cytotoxicity, DC2.4 cells were treated with MP_1000_-LPX according to the transfection procedure. Cell viability was investigated using an Apoptosis Detection Kit according to the manufacturer’s protocol by flow cytometry. Three independent cytotoxicity assays were performed in duplicate.

### Cellular Uptake of MP-LPX

Cellular uptake study was performed using Cou-6 as previously reported (Xu et al., 2016). DC2.4 cells in the logarithmic growth period were collected and seeded at a density of 8 × 10^5^ cells/well in a 24-well plate and incubated for 24 h at 37°C. To screen the appropriate incubation time, DC2.4 cells were treated with LPX (Cou-6, 5 ng/mL) in triplicate by different time from 0.5 h to 6 h. At the end of the study, the cells were collected and washed three times with cold phosphate buffer saline. The MFI of cells was quantified by BD FACS. To further screen the uptake concentration of Cou-6, DC2.4 cells were treated in triplicate by different concentrations of Cou-6 from 2.5 μg/mL to 20 μg/mL for 2 h.

To investigate the MR mediate uptake of MP_1000_-LPX (Cou-6, 10 ng/mL), DC2.4 cells were pre-treated with or without 0.16 mol/L of mannose solution for 30 min followed by incubation with LPX and MP_1000_-LPX at 37°C, respectively. For binding assays, DC2.4 cells were incubated at 4°C for 30 min. Subsequently, LPX and MP_1000_-LPX were added and incubated for 2 h at 4°C.

### Statistical Analysis

The data were presented as mean ± SEM unless otherwise noted. Statistical analysis was performed using Graphpad Prism 5.0. Data of two or multiple groups were analyzed using Student’s *t*-test or non-parametric one-way ANOVA, respectively. The *p*-values < 0.05 were considered statistically significant.

## Results

### Characterization of MP_n_-CH

#### Characterization of MP_100_-CH

**FIGURE 2:**
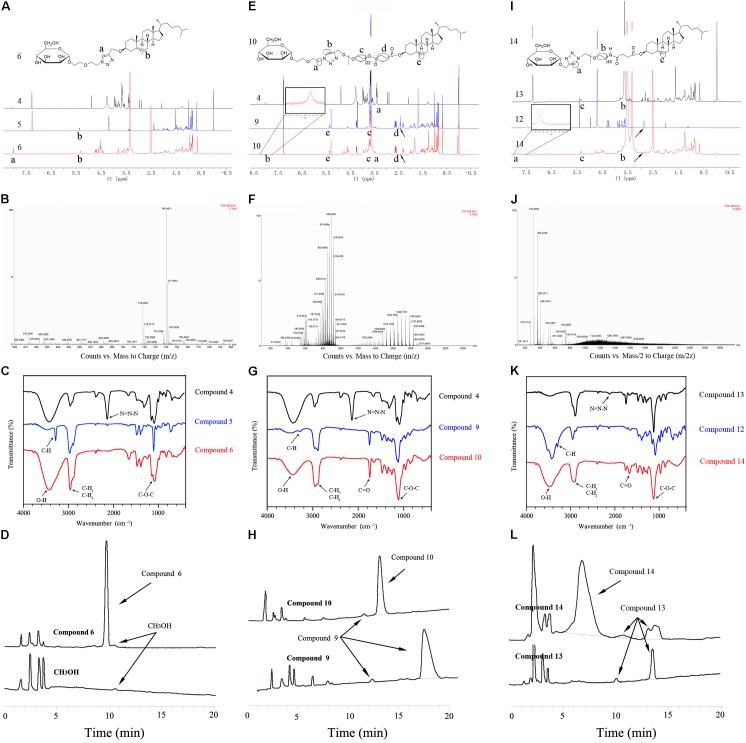
Characterization of MP_n_-CHs. **(A)** Comparison of the ^1^H NMR spectra of compounds **4**, **5**, and **6**. The protons of olefinic bond (N-CH = C-N) in coupled places were shown in compounds 6. **(B)** Mass spectrum of MP_100_-CH was shown as (M + Na)^+^, m/z: 740.4911. **(C)** FTIR spectrum of compounds **4**, **5**, and **6**. **(D)** High purity (97.984%) of MP_100_-CH was confirmed using HPLC. **(E)** Comparison of the ^1^H NMR spectra of compounds **4**, **9**, and **10** in CDCl_3_. **(F)** Mass spectrum of compounds 10 was shown as (M+Na)^+^, m/z: 1484.8359 to 1830.8468. **(G)** FTIR spectrum of compounds **4**, **9**, and **10**. **(H)** HPLC spectra was used to evaluate the purity of MP_1000_-CH (95.621%). ^1^H NMR spectra **(I)** and FTIR spectrum **(K)** of compounds **12**, **13**, and **14**. **(J)** Mass spectrum of MP_2000_-CH was shown as (M/2+Na)^+^, m/z. **(L)** The purity of MP_2000_-CH was evaluated (91.532%).

We construct MP_100_-CH (compound **6**) according to reasonable design as shown in **Figure 1A**. The structure of compounds **1**, **2**, **3**, **4**, and **5** were validated in **Supplementary Figures S1**–**S5**, respectively. As shown in **Figure 2A**, the ^1^H NMR spectra of compounds **4, 5** and **6** were recorded. The single peaks at δ5.33 (s) were attributed to the protons of olefinic bond (-CH_2_-CH = C-) in cholesterol. The single peak at δ8.01 (s) came from the protons of olefinic bond (N-CH = C-N) in coupled places. The peaks at δ3.15-3.88 (m) were attributed to the protons from the glycol unit (-O-CH_2_-CH_2_-O–CH_2-_) in PEG chain. These results indicated that MP_100_-CH has been successfully synthesized. As seen in **Figure 2B**, the mass spectrum of MP_100_-CH showed a peak at 740.49 (product + Na^+^), which was consistent with the expected molecular weight of MP_100_-CH. In addition, compound **6** was further confirmed by FTIR spectroscopy with of the following principal peaks: ν-OH (3700–3400 cm^-1^), ν-CH_3_ and ν-CH_2_ (2960–2850 cm^-1^), ν-CH_2_-O-CH_2_- (1210–1050 cm^-1^) presence but ν-N = N-N- (2100–2270 cm^-1^) attributed to compound 4, ν-CH (about 3300 cm^-1^) attributed to compound 5 absence in compounds **6** (**Figure 2C**). Finally, as shown in **Figure 2D**, the spectrum of HPLC exhibited a characteristics absorption peak of MP_100_-CH at 9.743 min (201 nm). According to the method of area normalization, the purity of MP_100_-CH is 97.948%.

#### Characterization of MP_1000_-CH

To construct MP_1000_-CH (compound **10**), compound 4 was jointed to compound 9 via click reaction as shown in **Figure 1B**. The structure of compounds **8** and **9** were firstly confirmed in **Supplementary Figures S6**, **S7**, respectively. The ^1^H NMR spectra of compounds **4, 9** and **10** were recorded in CDCl_3_. The principal proton peaks at δ2.42-2.45 (t) attributed to the protons of alkynyl group (-CH) in compounds **9** were disappeared in compounds **10** as in shown **Figure 2E**. Similar to MP_100_-Chol (presence of single peak at δ8.01), the successful synthesis of MP_1000_-Chol has also been validated. The mass spectrum of MP_1000_-Chol showed broad peaks from 1484.8359 to 1830.8468 (**Figure 2F**) while that of compound 9 were from 1185.8085 to 1480.0358 (**Supplementary Figure S8**). The increased molecular weight coincided with the molecular weight of compounds **4**, which also confirmed the structure of compound 10 referring to previously report (Li et al., 2014). The FIIR spectroscopy of compounds 10 (**Figure 2G**) was similar to compounds 4 but the presence of unique ν-CH_2_-CO-O- of compounds 10 at around 1740 cm^-1^. Additionally, the characteristics absorption peak (201 nm) of compounds **10** and compounds **9** were at 13.122 min with the purity of 96.595% (**Figure 2H**) and 16.692 min with the purity of 94.881%, respectively. Consistent with expectation, the hydrophilicity of compound 10 was increased and the retention time was decreased when compared with compound 9. All of the results of ^1^H NMR spectra, mass spectrum, FTIR spectroscopy and HPLC confirmed the successful synthesis of compounds **10**.

#### Characterization of MP_2000_-CH

Similar to compound **10**, compound **14** was acquired according to our designed strategies (**Figure 1C**) and authenticated via ^1^H-NMR (**Figure 2I**), electrospray ionization mass spectrometry (ESI-MS) (**Figure 2J**), FTIR (**Figure 2K**), and HPLC (**Figure 2L**). The structure of compounds **11** and **12** were firstly confirmed in **Supplementary Figures S9**, **S10**, respectively. Of note, the measured molecular weight of compound **12** was 241.07 (product + Na^+^) (**Supplementary Figure S11**), which was consistent with the expected molecular weight. When compared mass spectrum of compound 14 (**Figure 2J**) with compound 13 (**Supplementary Figure S12**), nearly 200 molecular weight were increased. The retention time at 201 nm of compound 14 and compound 13 were at 6.907 min with the purity of 91.532% and 13.662 min with the purity of 91.251% (**Figure 2L**), respectively.

### Particle Size and Zeta Potential Measurement

**FIGURE 3:**
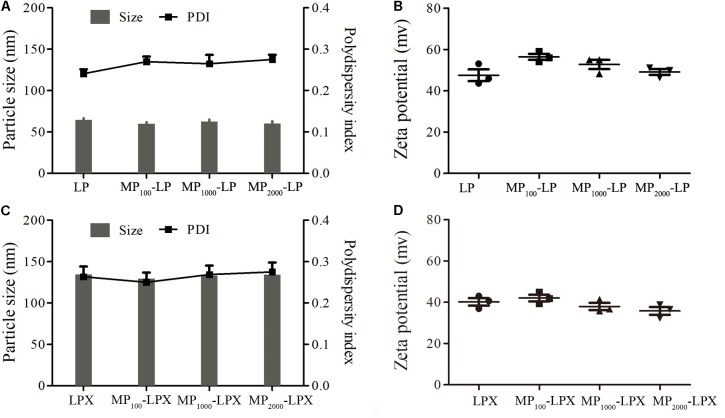
Physicochemical properties of MP_n_-LPs and MP_n_-LPX. Size **(A)** and zeta potential **(B)** of MP_n_-LPs with different PEG linkers. Size **(C)** and zeta potential **(D)** of different formulations loaded with mRNA.

The size and zeta potential of all formulations in this study were evaluated. There was no statistical difference between the particle size and zeta potential among different MP_n_-LPs or MP_n_ LPX formulations. The particle size and zeta potential of all different MP_n_-LPs was about 60 nm (**Figure 3A**) and 50 mV (**Figure 3B**), respectively. In addition, the particle size and zeta potential of all different LPX was about 135 nm (**Figure 3C**) and 40 mV (**Figure 3D**), respectively. The PDI were all less than 0.3. As was shown, the size of MP_n_-LPX was larger and the zeta potential was lower than the corresponding MP_n_-LPs formulations.

### *In vitro* Transfection of MP_n_-LPX

**FIGURE 4:**
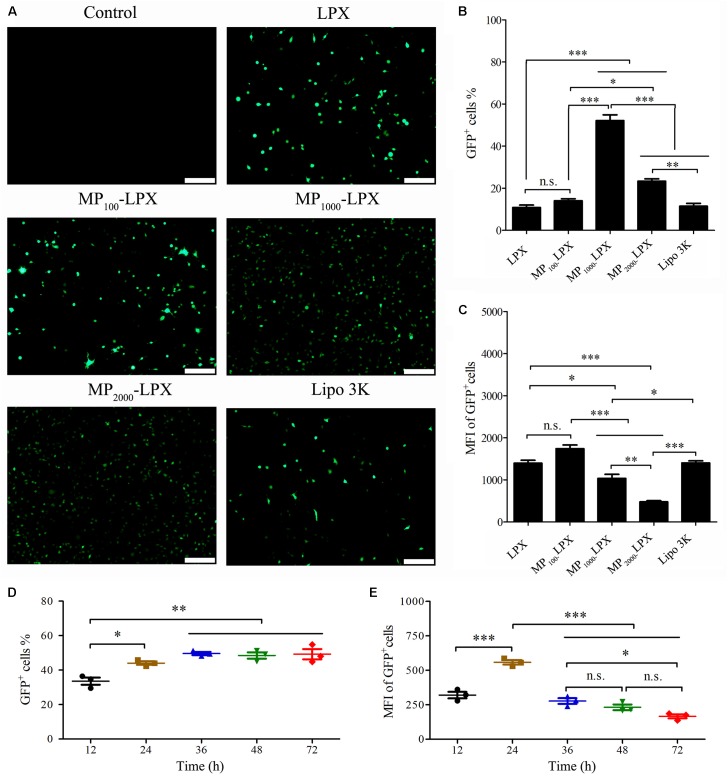
Transfection of MP_n_-LPX with different PEGs length on DC2.4 cells at N/P 5. **(A)** GFP expression by DC2.4 cells observed by a fluorescence microscope. Transfection efficiency (%GFP^+^ cells) **(B)** and MFI of GFP^+^ cells **(C)** were quantified. Kinetics of MP_1000_-LPX NP 5 transfection efficiency **(D)** and MFI of GFP positive cells **(E)** on DC2.4 cells from 12 h to 72 h. Scale bars, 200 μm. ^∗^*p* < 0.05; ^∗∗^*p* < 0.01; ^∗∗∗^*p* < 0.001.

To investigate the *in vitro* transfection efficacy of MP_n_-LPX in DC2.4 cells, the appropriate ratio of N/P of LPX was optimized firstly. GFP expression on DC2.4 cells with different treatment were observed and recorded by fluorescence microscope (**Supplementary Figures S13A–H**). As shown in **Supplementary Figure S13I**, both LPX NP 7 and LPX NP 5 achieved significant increment in transfection efficiency compared with LPX NP 3. What’s more, LPX NP 5 with similar transfection efficiency to LPX NP 7 exhibited dramatically enhanced GFP fluorescence intensity (mean GFP expression level per cell) (**Supplementary Figure S13J**). By the way, calculation of MFI and GFP positive cells was shown in **Supplementary Figure S14**. Thus, MP_n_-LPX were prepared by setting the N/P ratio at 5:1. The GFP expression was subsequently observed by fluorescence microscope (**Figure 4A**). As shown in **Figure 4B**, MP_1000_-LPX induced the most GFP positive cells and the percentage was up to 52%, which was significantly higher than any other groups (*p* < 0.001). However, the MFI of the GFP positive cells of MP_1000_-LPX NP 5 was not the best among these groups (**Figure 4C**). Taking into account of transfection efficiency and MFI, MP_1000_-LPX with the highest transfection efficiency and moderate MFI were selected for further study in this manuscript.

The kinetics of MP_1000_-LPX NP 5 transfection on DC2.4 cells was studied. As shown in **Figures 4D,E**, transfection efficiency first increased and then reached a plateau with the increase of the incubation time (from 12 to 72 h) while the MFI of GFP positive cells first increased and then decreased. In summary, the transfection efficiency achieved the maximum at 24 h and MFI was also the strongest at 24 h.

### Further Study on the Optimal MP_1000_-LPX

#### Morphology Examination

**FIGURE 5:**
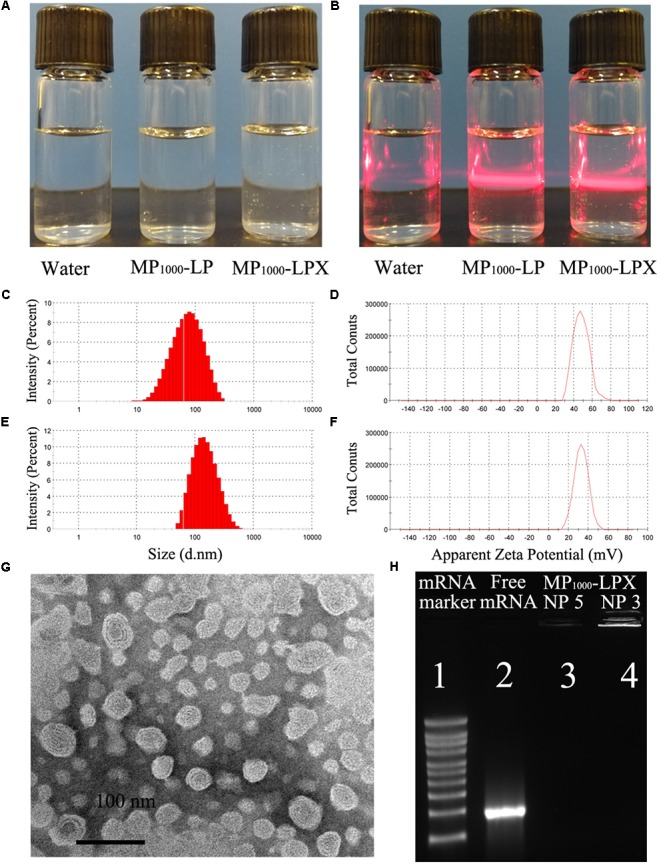
Characterization of MP_1000_-LPX. Appearance **(A)** and Tyndall effect **(B)** of MP_1000_-LPX. Size **(C)** and zeta potential **(D)** of MP_1000_-LP were recorded by Zetasizer Nano ZS90, and representative images were showed. Representative images of size **(E)** and zeta potential **(F)** of MP_1000_-LPX. **(G)** TEM images of the MP_1000_-LPX NP 5. **(H)** Agarose gel electrophoresis image of mRNA maker, Free mRNA, MP_1000_-LPX (N/P 5) and MP_1000_-LPX (N/P 3).

The appearance and morphological studies of MP_1000_-LPX were conducted. The colloidal solution was colorless and transparent (**Figure 5A**). Overt Tyndall effect of MP_1000_-LPs and MP_1000_-LPX colloidal solution were observed compared with water as was shown in **Figure 5B**. Representative images of size and zeta potential of MP_1000_-LP(X) were shown in **Figures 5C–F**, respectively. As shown in **Figure 5G**, the morphological of MP_1000_-LPX was observed distinct lipid membrane structure with nearly spherical in shape. Moreover, complete complexation of the mRNA with MP_1000_-LPs was confirmed when the N/P ratio of 3 and 5 (**Figure 5H**).

#### Stability Assessment

**FIGURE 6:**
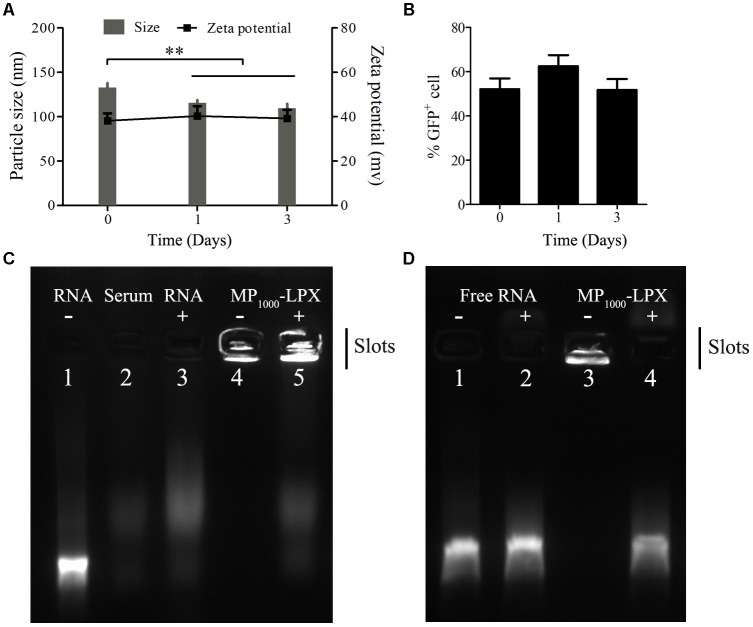
Stability of MP_1000_-LPX. **(A)** Size and zeta potential measurements of MP_1000_-LPX at 0, 1, and 3 days stored at 4°C. **(B)** Transfection efficiency of MP_1000_-LPX at 0, 1, and 3 days stored at 4°C. **(C,D)** Gel electrophoresis retardation assay of MP_1000_-LPX (N/P:5:1) to test serum stability. **(C)** Free RNA (lane 1), Serum (lane 2), Serum + RNA (lane 3), MP_1000_-LPX (lane 4) and Serum + MP_1000_-LPX (lane 5). **(D)** Free RNA in 150 mM NaCl treated without (lane 1) or with Triton X-100 (lane 2). MP_1000_-LPX in 150 mM NaCl treated without (lane 3) and or with Triton X-100 (lane 4). ^∗∗^*p* < 0.01.

The preliminary storage stability of the MP_1000_-LPX was determined by the size, zeta potential and transfection efficiency. The particle size and zeta potential of MP_1000_-LPX were determined at predetermined time of storage at 4°C. MP_1000_-LPX displayed a little decrease in particle size but not zeta potential (**Figure 6A**) As shown in **Figure 6B**, the transfection efficiency of MP_1000_-LPX remained about 50% when stored at 4°C for 3 days. Additionally, MP_100_-LPX (**Supplementary Figure S15A**) and MP_2000_-LPX (**Supplementary Figure S15B**) performed excellent storage stability in the preliminary test.

For serum stability, 5 μL of fresh MP_1000_-LPX were diluted in FBS (1:1, v/v) and incubated for 2 h at 37°C. As shown in **Figure 6C**, the signal of naked mRNA band in serum was completely disappeared (lane 3) compared to naked mRNA alone (lane 1). MP_1000_-LPX did not dissociate after incubation in 50% serum (lane 5) similar to that incubation in NaCl of 150 mM (lane 4). When the free mRNA or MP_1000_-LPX were treated with Triton X-100, the free mRNA (lane 2) and mRNA dissociated from MP_1000_-LPX (lane 4) were visible in line with mRNA treated without Triton X-100 (lane 1) as shown in **Figure 6D**. Similarly, MP_100_-LPX and MP_2000_-LPX exerted good stability in the presence of serum (**Supplementary Figure S15C**). These results confirmed the adequate protection of the mRNA against degradation.

#### Cytotoxicity Assay

**FIGURE 7:**
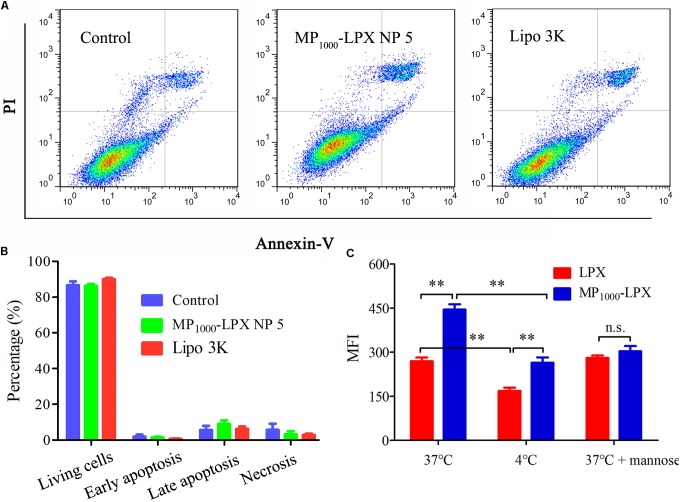
Cytotoxicity and uptake assay of MP_1000_-LPX on DC2.4 cells. **(A,B)** Flow cytometry measurement of cell apoptosis induced by MP_1000_-LPX NP 5 and Lipo3K on DC2.4 cells. The data showed mean ± SEM from a representative experiment from three independent experiments. **(C)** Cellular uptake mediated by mannose receptor at different conditions, including incubated at 4, 37, or 37°C with free mannose. ^∗^*p* < 0.05; ^∗∗^*p* < 0.01.

After incubation with indicated formulations for 24 h, cytotoxicity analysis was performed by flow cytometry. Representative figure of each condition was showed in **Figure 7A**. The percentage of living DC2.4 cells were found to be 86.7 ± 3.6 %, 86.4 ± 1.7%, and 90.1 ± 1.2% (**Figure 7B**) for control (treated with equal volume of medium), MP_1000_-LPX and Lipo 3K group, respectively. None significant percentage difference of living cells, early apoptosis, late apoptosis or necrosis was found among these three groups indicating that MP_1000_-LPX might do no harm to DC2.4 cells. Overall, MP_1000_-LPX NP 5 performed good safety *in vitro*. Considering the excellent transfection efficacy, MP_1000_-LPX might be one of good candidates for DC-targeting mRNA nanovaccine for *in vivo* application.

### *In vitro* Uptake

In the preliminary uptake experiment, incubation time and concentration of Cou-6 were optimized. According to the results in **Supplementary Figures S16A,B**, 2 h and 10 ng/mL were selected for future cellular uptake experiment, respectively. To evaluate the potency of MP_1000_-LPX NP 5 on targeted delivery into DC2.4 cells, the fluorescent of Cou-6 internalized by cells was assayed by BD FACS. The results in **Figure 7C** indicated that intracellular uptake of MP_1000_-LPX was significantly higher than that of LPX at 37 and 4°C, respectively. When pretreated the cells with mannose, no significant difference of Cou-6 in cellular uptake between MP_1000_-LPX and LPX was observed. Moreover, intracellular uptake of LPX and MP_1000_-LPX was significantly lower at 4°C than that at 37°C, indicating that uptake of LPs loaded with mRNA was energy dependent.

## Discussion

In the presented study, we designed facile and inexpensive approach to prepare mannose-cholesterol conjugates with various linker length as synthetic ligands applied to mRNA nanovaccine. MP_n_-LPs were prepared by a modified thin-film dispersion method. Subsequently, the DCs targeting MP_n_-LPX were prepared by complexing MP_n_-LPs with mRNA. No significant difference in size and zeta potential was observed among MP_n_-LPX comprised MP_n_-CH with different PEG units. The effect of linker length of mannose derivatives in MP_n_-LPX on transfection by DC2.4 cells was investigated. Our results might provide a rational design element of mRNA vaccine.

Linker length of ligand exerted significantly effect on targeting cellular uptake (Engel et al., 2003; Stefanick et al., 2013; Jeong et al., 2014). Thus, a proper linker length of the ligands was essential for effective receptor recognize and binding. For recognition and binding to MR, linker length of mannose should consist of at least two PEG units according to previously report (Jeong et al., 2014). In our study, mannose-cholesterol conjugates with different linker length were designed and constructed by facile strategies utilizing the click reaction. In detail, mannose derivatives have been conjugated to cholesterol derivatives modified with PEG of different lengths (PEG_100_, PEG_1000_, or PEG_2000_). Each target product was fully characterized by ^1^H-NMR, ESI-MS, FTIR to confirm the successful of synthesis. HPLC was used to evaluate the purity and the successful synthesis of the products as previously reported (Shariat et al., 2014). The synthetic strategies designed here offered some overt advantages over the previously reported methods for Man-C_4_-chol (Kawakami et al., 2000) and Man-C_6_-chol (Li et al., 2013), because our target products were easily to synthesis, purify and characterize. What’s more, the length of PEG-linkers could be varied with desired length, resulting in many other analogous compounds with MR targeting function.

We constructed MP_n_-LPs with various linker lengths between cholesterol and mannose using different length of PEG linker. All MP_n_-LPs were constructed with the same molar ratio of mannose modified cholesterol. The MP_n_-LPs had similar average size and surface charge despite of the introduction of MP_n_-CHs with different linker lengths of PEG. We then prepared MP_n_-LPX and performed cellular transfection studies in DC2.4 cells with the evaluated parameters of transfection efficiency and MFI of GFP positive cells, respectively. Transfection efficiency and MFI of LPX was preliminary optimized to find optimal ratio of N/P. Our results showed no significant difference in transfection efficiency of LPX NP 5 and LPX NP 7, while both exhibited significantly higher transfection efficiency than LPX NP 3. However, MFI of the GFP positive cells of LPX NP 5 was significantly higher compared to that of LPX NP 7. Taken the transfection efficiency and MFI of the GFP positive cells into consideration, the ratio of N/P of 5 was selected for future studies. The difference between the percentage of transfected cells and the MFI of transfected cells was consistent with previously reported results (McLenachan et al., 2013; Avci-Adali et al., 2014; Lee et al., 2015; Li et al., 2017a). Transfection using the same transfection reagent led to similar transfection efficiency but not the MFI (**Figures 4D,E**) with the increase of the incubation time, which was consistent with previously report (Avci-Adali et al., 2014). In addition, increasing the amount of mRNA can significantly increase the average fluorescence intensity without affecting the transfection efficiency within a certain range (Avci-Adali et al., 2014). Moreover, MFI values showed the strength of the fluorescence intensity. Higher MFI values reflected a higher production of GFP by individual cell but not higher percentage of GFP positive cells.

According to literatures, upon interaction with serum, nanocarriers rapidly absorbed protein and formed a corona (Bertrand et al., 2017; Pan et al., 2017). It was the nanocarrier–corona complex, rather than the nanocarrier, that interacted with biological systems, here with a cell membrane receptor (CD206), which might partially obscure the role of target ligands (Monopoli et al., 2012). To reduce this effect, culture medium without serum was used at initial and complete culture medium with serum were added 4 h after the adding of LPX. As was known, the fate of nano-preparations in serum (mimic the *in vivo* environment) was very important to predict its potential therapeutic efficacy. Our preliminary test showed that the presence serum significantly affected the transfection of MP_1000_-LPX. We were still working on this. Hopefully, we would show the data of mRNA delivery in the presence of serum *in vitro* and expression of the nano-preparations *in vivo* in our future work.

MP_1000_-LPX exhibited a higher level of transfection efficiency than LPX, MP_100_-LPX, MP_2000_-LPX and positive control Lipo 3K. No size and charge variation were found in the vectors then no inference can be made on the transfection efficiency correlation with size and charge. The excellent transfection efficiency of MP_1000_LPX was most likely attributed to the appropriate linker length used to conjugate mannose and cholesterol. However, MP_1000_LPX exerted the highest transfection efficiency but moderate MFI. The inconsistency of cellular transfection efficiency and MFI observed in the field of targeted mannose modified LPX partially resulted from differences in the types of cell, incubation time, the amount of mRNA, ability of lysosome escape and types of lipid mannose modified (Kim et al., 2012; Li et al., 2013; Avci-Adali et al., 2014; Chen et al., 2015; Wang C. et al., 2015). The best effect of MP_1000_-LPs conjugates may be related to the above factors. However, most likely it was attributed to the linkers used to conjugate mannose and the other different component of targeting formulations (Kim et al., 2012; Wang N. et al., 2014; Wang C. et al., 2015). There are many other known and unknown factors for ligand receptor affinity beside linker length of ligand. Moreover, the intracellular metabolism of mRNA nanovaccine might also affect the expression of protein encoded by mRNA. Accurately, we cannot declare that the linker length of MP_1000_-LPX is optimal for transfection by DC2.4 cells but selected MP_1000_-LPX as a representative formulation from our result for further investigation.

The pharmaceutical properties including particle size, zeta potential, storage stability and the ability to protect mRNA against serum degradation of optimal MP_1000_-LPX were then characterized systemically. MP_1000_-LPX displayed bigger diameter and lower zeta potential compared to MP_1000_-LPs, indicating the complexation of MP_1000_-LPs with mRNA. We were surprised to find that particle size measurement results of MP_1000_-LPX by Zetasizer Nano ZS90 was much larger than that by TEM although some other researchers also observed the similar phenomenon (Wang K. et al., 2018; Yang et al., 2018). The larger size distribution by Zetasizer than TEM observed in the field of size measurement partially resulted from the interference of the dispersant into the hydrodynamic diameter. Complete complexation of the mRNA with MP_1000_-LPs was also validated according to the results of gel electrophoresis retardation assay. The preliminary storage stability experiment revealed that MP_1000_-LPX could maintain its excellent transfection efficiency at least for 3 days at 4°C, which might benefit from the protection of the mRNA against degradation. It has been reported that triMN-LPR with high zeta potential (about 35 mV) could better target human and murine dendritic cells, result in higher recruitments of DCs to draining lymph nodes, and induced significant antitumor responses (Le Moignic et al., 2018). Additionally, Folate modified cationic LPs loaded with DNMT1 gene with positive charge (>30 mV) exerted excellent *in vitro* targeted genome editing and *in vivo* antitumor effects (He et al., 2018). However, there seemed to be much cationic charge in the MP_1000_-LPX. Further formulation optimization will be done to balance the transfection efficiency and the high positive zeta potential in our future work.

The cellular cytotoxicity and uptake mechanism of MP_1000_-LPX were also evaluated. MP_1000_-LPX presented good safety *in vitro* according to the data of cell apoptosis and might be a safe formulation for *in vivo* application. It has been reported that the presence of free mannose could decrease the uptake of mannose modified preparations (Li et al., 2013; Wang C. et al., 2014). The amount of free mannose in this study was used according to a previously reported literature (Wang C. et al., 2014). Moreover, the experiment design was similar to previous reports (Li et al., 2013; Wang C. et al., 2014). When DC2.4 cells were pretreated with free mannose as an inhibitor, no significant effect on uptake by LPX was observed, while uptake by MP_1000_-LPX was significantly decreased. This difference in uptake indicated that the enhanced uptake and transfection were mainly through the MR on DC2.4 cells in line with previous reports (Li et al., 2013; Wang C. et al., 2014). Taken together, our results of uptake *in vitro* confirmed that the enhanced transfection of MP_1000_-LPX occurs mainly via a MR-mediated mechanism and the linker length of mannose exerts a crucial role. Although MP_1000_-LPX exhibited higher level of transfection efficiency through the MR than MP_100_-LPX and MP_2000_-LPX, we could not exclude that other linker length of mannose modified cholesterol would exhibit more effective transfection in DC2.4 cells through MR. Nevertheless, a rational design element was proposed and more detailed future studies will be indispensable to facilitate the progression of mRNA nanovaccine.

## Conclusion

In summary, MP_n_-CHs with different linker molecules (PEG_100_, PEG_1000_, and PEG_2000_) were successfully synthesized by a simple and cost-efficient method. The DC-targeting LPs complexed with mRNA were self-assembled using MP_n_-CHs as the targeting lipids. The linker molecules had no effect on the particle size and zeta potential of LPs and mRNA-complexed LPs but significantly affected the transfection efficiency of GFP-encoding mRNA. Unexpectedly, PEG_1000_ rather than PEG_100_ or the commonly used PEG_2000_ as the linker achieved the maximal level of GFP expression. MP_1000_-LPX containing MP_1000_-CH displayed good profiles including small size with nearly spherical shape, good stability in serum and little cytotoxicity, indicating a hopeful DCs-targeting delivery system for mRNA vaccine.

## Author Contributions

XS conceived the project. MF and JW designed the experiments. FW, WX, and XB conducted most of the experiments. WX and QZ further performed and analyzed the transfection and characterization of formulations. FW, ME, and WX drafted the manuscript. LG and SY performed the ^1^H-NMR and HPLC analysis. QZ and YZ performed the some preliminary experiments. WX, QZ, and AF participated in literature searching. XS and MF finished the manuscript editing. All authors reviewed and approved the manuscript.

## Conflict of Interest Statement

The authors declare that the research was conducted in the absence of any commercial or financial relationships that could be construed as a potential conflict of interest.
