# Supplementary material for: Optimization of the Linker Length of Mannose-Cholesterol Conjugates for Enhanced mRNA Delivery to Dendritic Cells by Liposomes
Source: Front Pharmacol. 2018 Sep 5;9:980. doi: 10.3389/fphar.2018.00980 (PMC6134263; doi:10.3389/fphar.2018.00980)
Supplement: Supplementary file 1 [file Data_Sheet_1.pdf]

Supplementary Materials

Optimization of the linker length of mannose-cholesterol conjugates for enhanced mRNA delivery to dendritic cells by liposomes

Fazhan Wang[1, †], Wen Xiao[1, †], Mostafa A. Elbahnasawy[2, †], Xingting Bao[1], Qian Zheng[1], Linhui Gong[1], Yang Zhou[1], Shuping Yang[1], Aiping Fang[1], Mohamed M. S. Farag [2, *], Jinhui Wu[1, *], Xiangrong Song[1, *]

[1]State Key Laboratory of Biotherapy, Geriatrics and Cancer Center, West China Hospital, Sichuan University, and Collaborative Innovation Center for Biotherapy, Sichuan University, Chengdu, China

[2]Botany and Microbiology Department, Faculty of Science, Al-Azhar University, Nasr City, Cairo, Egypt

[†] These authors contributed equally to this article.

[*] Corresponding author. State Key Laboratory of Biotherapy, Geriatrics and Cancer Center, West China Hospital, Sichuan University, and Collaborative Innovation Center for Biotherapy, Sichuan University, Chengdu 610041, China.

E-mail address: songxr@scu.edu.cn (X. Song); wujinhui@scu.edu.cn (J. Wu); mohamed.farag@azhar.edu.eg (M. Farag)

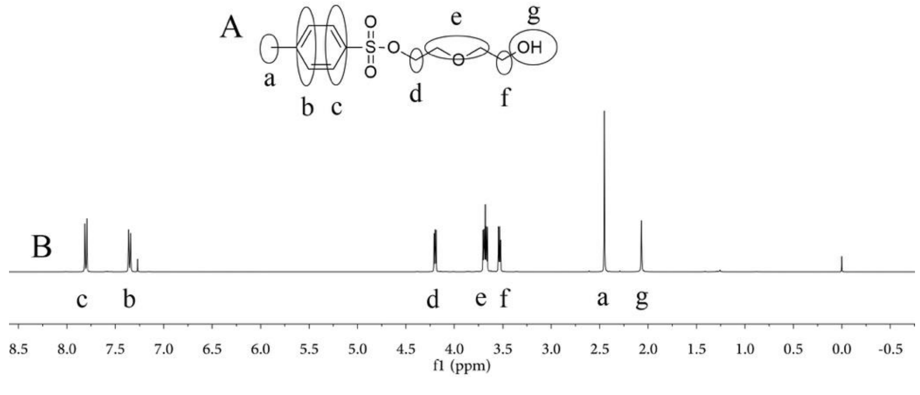

Figure S1 ^{1}H NMR spectra of compound **1**. (A) Structural formula of compound **1**. (B) ^{1}H NMR spectra of compound **1**.

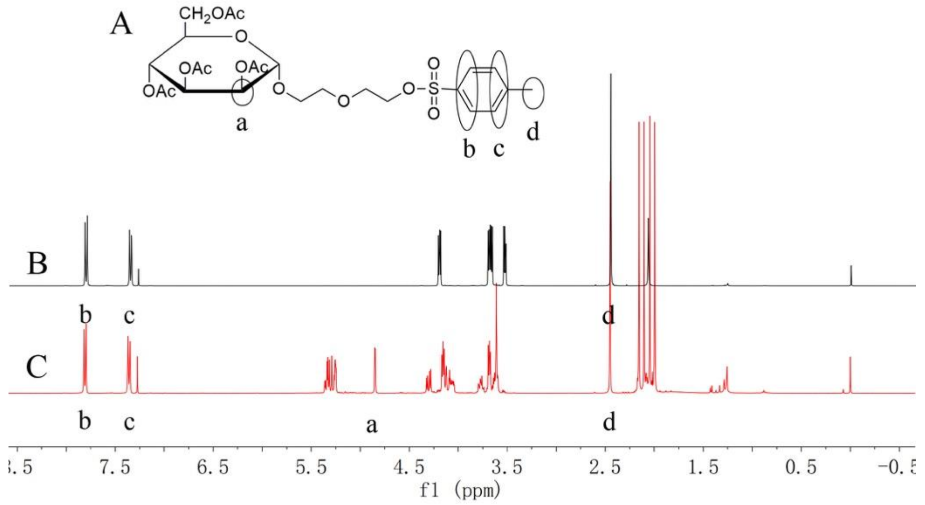

Figure S2 Comparison of the ^{1}H NMR spectra of compound **1** and compound **2**. (A) Structural formula of compound **2**. (B) ^{1}H NMR spectra of compound **1**. (C) ^{1}H NMR spectra of compound **2**. The principal proton peaks of compound **1** were shown in compound **2**. The multiple peaks at δ1.97-2.17(m) came from the protons of acetyl group (-OOCCH3) also shown in compound **2**.

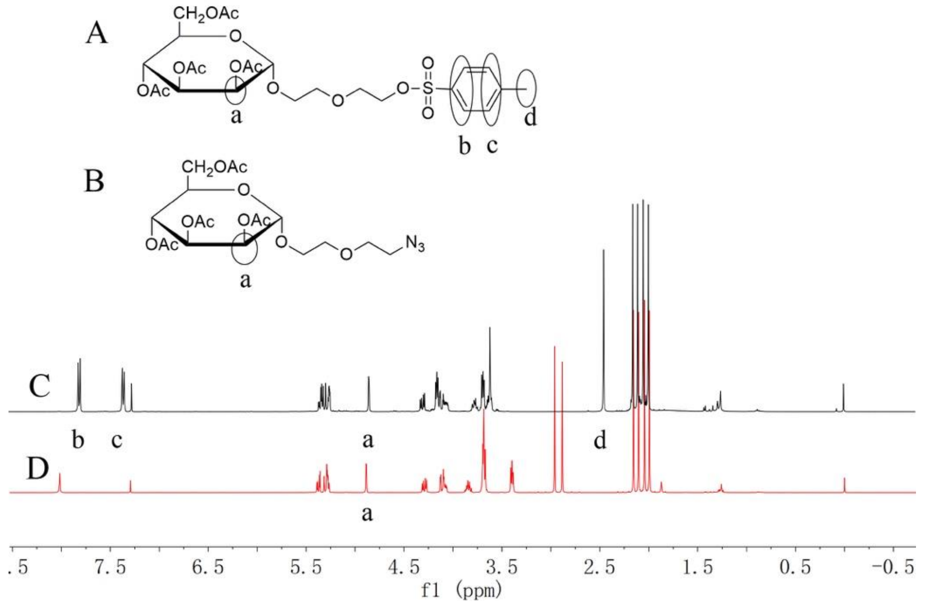

Figure S3 Comparison of the ^{1}H NMR spectra of compound **2** and compound **3**. (A) Structural formula of compound **2**. (B) Structural formula of compound **3**. (C) ^{1}H NMR spectra of compound **2**. (D) ^{1}H NMR spectra of compound **3**. The principal proton peaks at δ7.81(d) and δ7.36(d) attributed to the protons of benzene ring (-Tos) in compound **2** were disappeared in compound **3**.

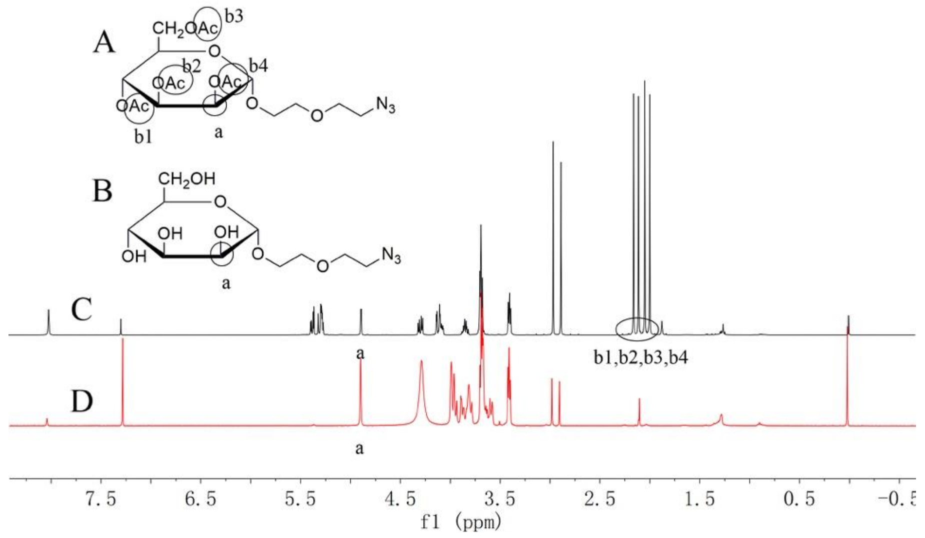

Figure S4 Comparison of the ^{1}H NMR spectra of compound **3** and compound **4**. (A) Structural formula of compound **3**. (B) Structural formula of compound **4**. (C) ^{1}H NMR spectra of compound **3**. (D) ^{1}H NMR spectra of compound **4**. The principal proton peaks at δ1.98-2.18 (dd) attributed to the protons of acetyl group (-OOCCH_3) in compound **3** were disappeared in compound **4**.

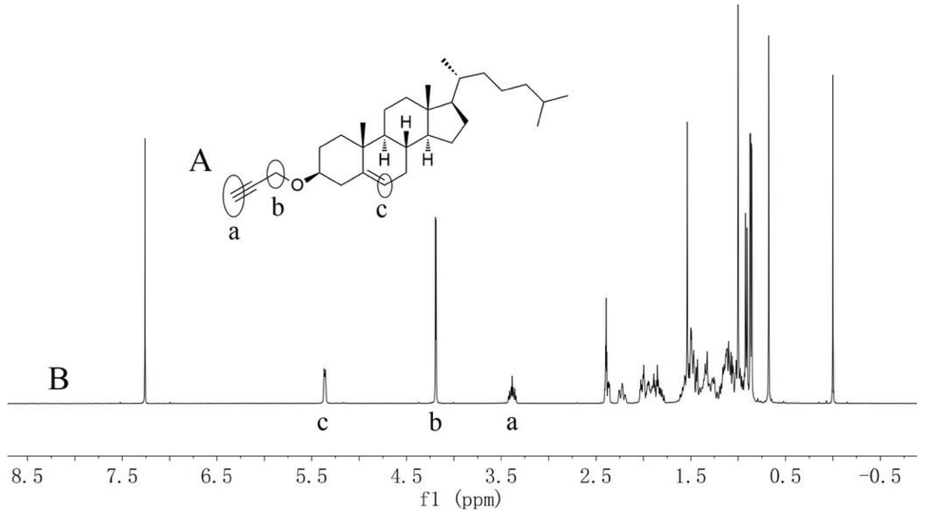

Figure S5 ^{1}H NMR spectra of compound **5**. (A) Structural formula of compound **5**. (B) ^{1}H NMR spectra of compound **5**.

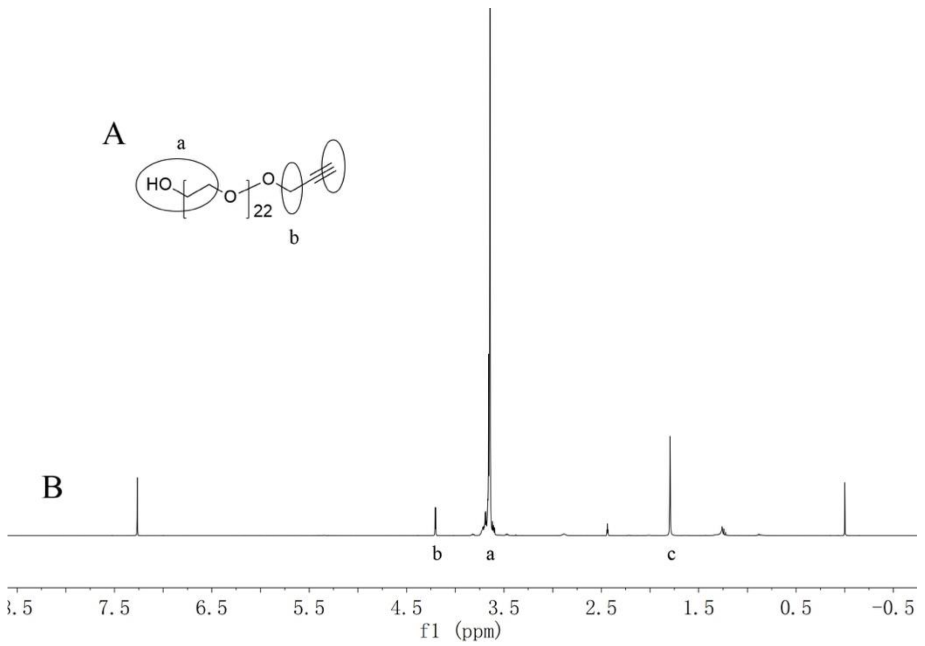

Figure S6 ^{1}H NMR spectra of compound **8**. (A) Structural formula of compound **8**. (B) ^{1}H NMR spectra of compound **8**.

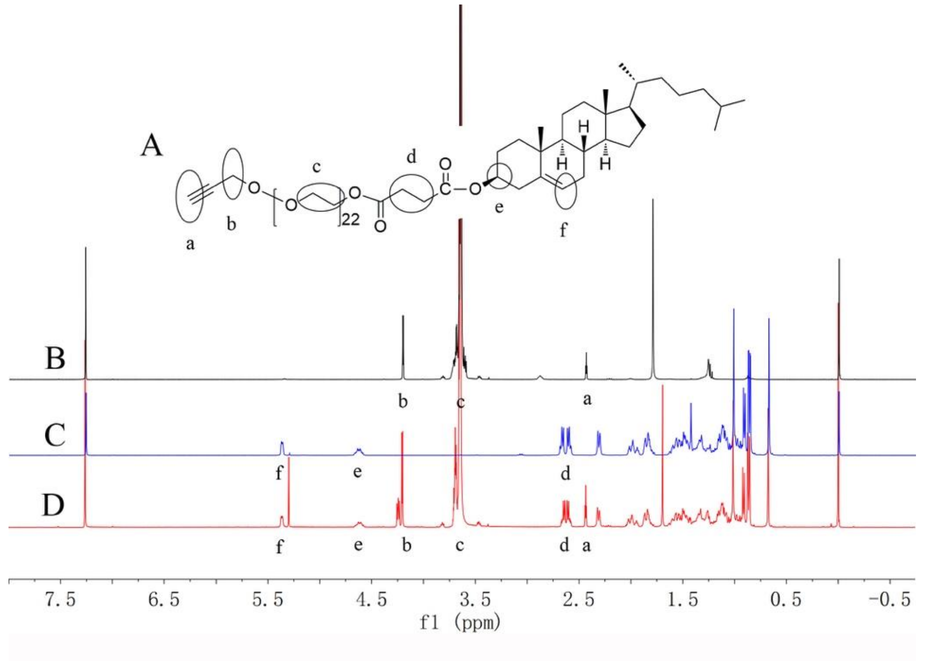

Figure S7 Comparison of the ^{1}H NMR spectra of compound **8**、compound **7** and compound **9**. (A) Structural formula of compound **9**. (B) ^{1}H NMR spectra of compound **8**. (C) ^{1}H NMR spectra of compound **7**. (D) ^{1}H NMR spectra of compound **9.** The principal proton peaks of compound **8** and compound **7** were shown in compound **9**.

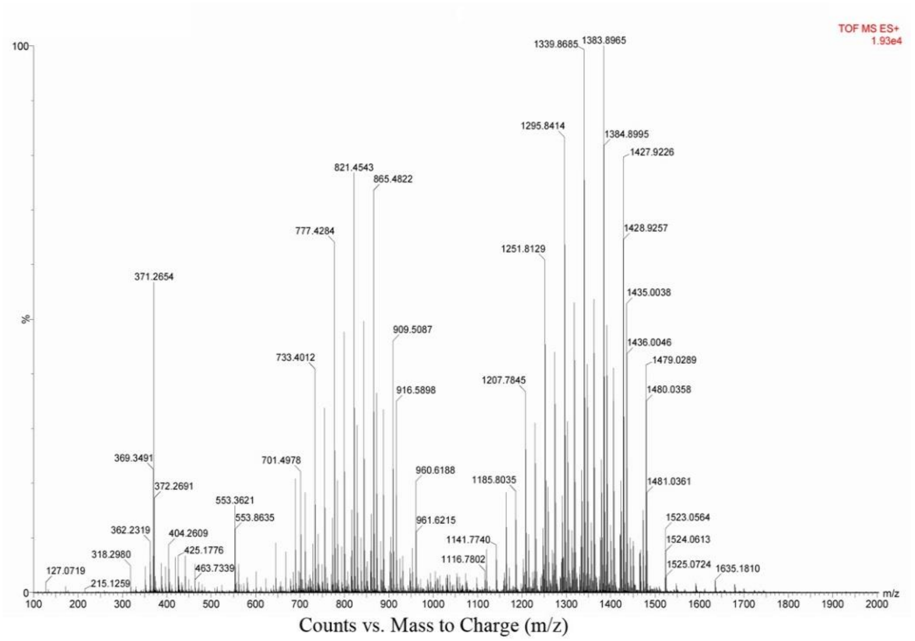

Figure S8 Mass spectrum of compound **9**. $(M+Na)^+$ m/z.

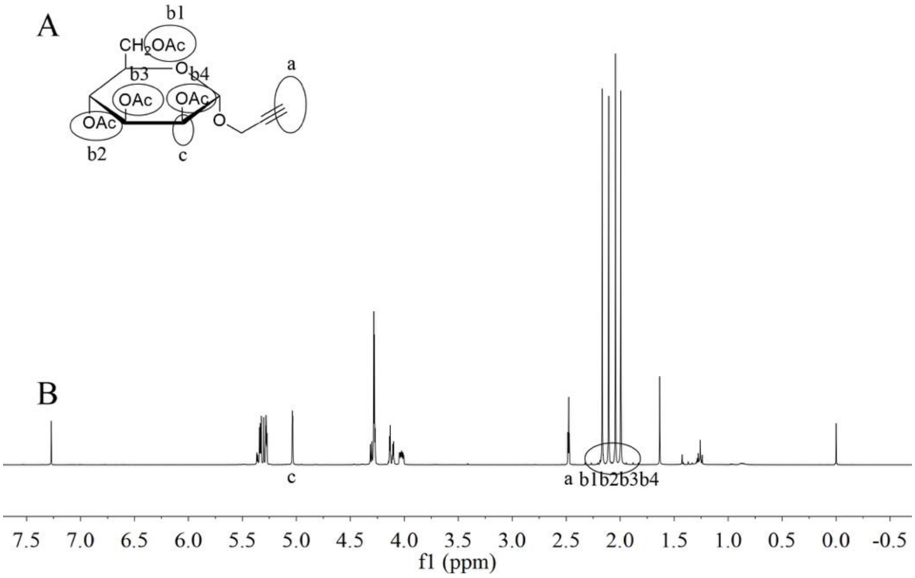

Figure S9 ^{1}H NMR spectra of compound **11**. (A) Structural formula of compound **11**. (B) ^{1}H NMR spectra of compound **11**.

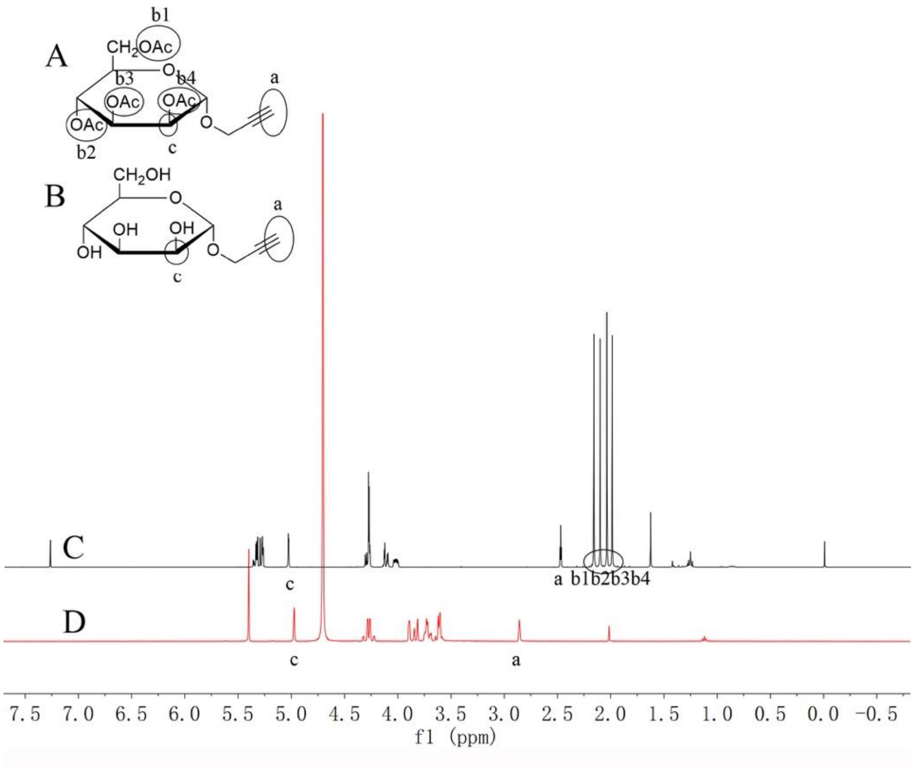

Figure S10 Comparison of the ^{1}H NMR spectra of compound **11** and compound **12**. (A) Structural formula of compound **11**. (B) Structural formula of compound **12**. (C) ^{1}H NMR spectra of compound **11**. (D) ^{1}H NMR spectra of compound **12**. The principal proton peaks at δ1.90-2.90 (m) attributed to the protons of acetyl group (-OOCCH$_3$) in compound **11** were disappeared in compound **12**.

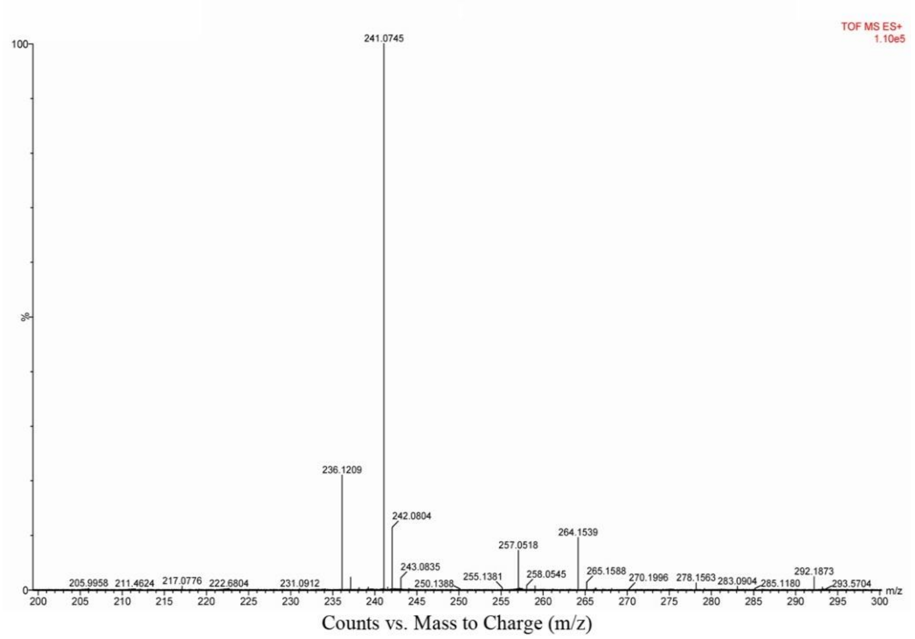

Figure S11 Mass spectrum of compound **12**. $(M+Na)^{+}$ m/z.

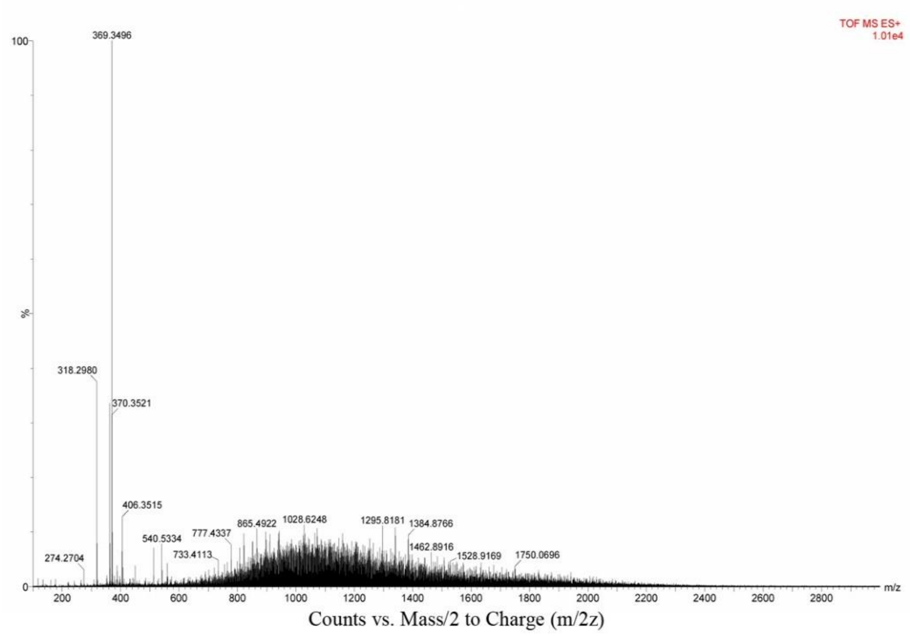

Figure S12 Mass spectrum of compound **13**. $(M/2+Na)^+$ m/2z.

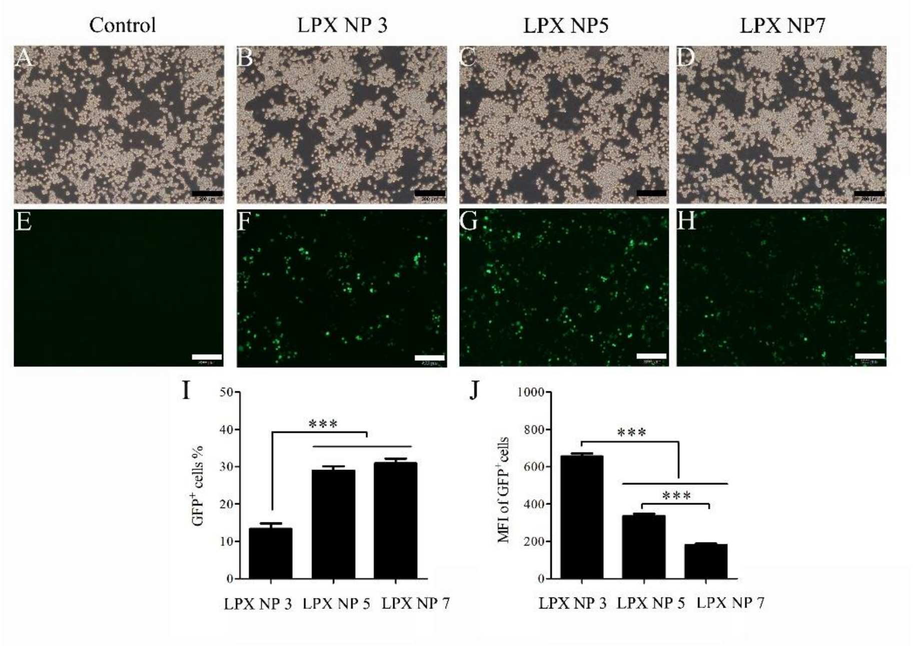

Figure S13 Transfection of LPX on DC2.4 cells at different N/P ratio. White light (A-D) and fluorescence (E-H) images of control, LPX NP 3, LPX NP 5 and LPX NP 7, respectively. Transfection efficiency (%GFP$^+$ cells) (I) and MFI of GFP$^+$ cells (J) of LPX NP 3, LPX NP 5 and LPX NP 7 quantified by flow cytometry. Scale bars, 200 μm. ***p <0.001.

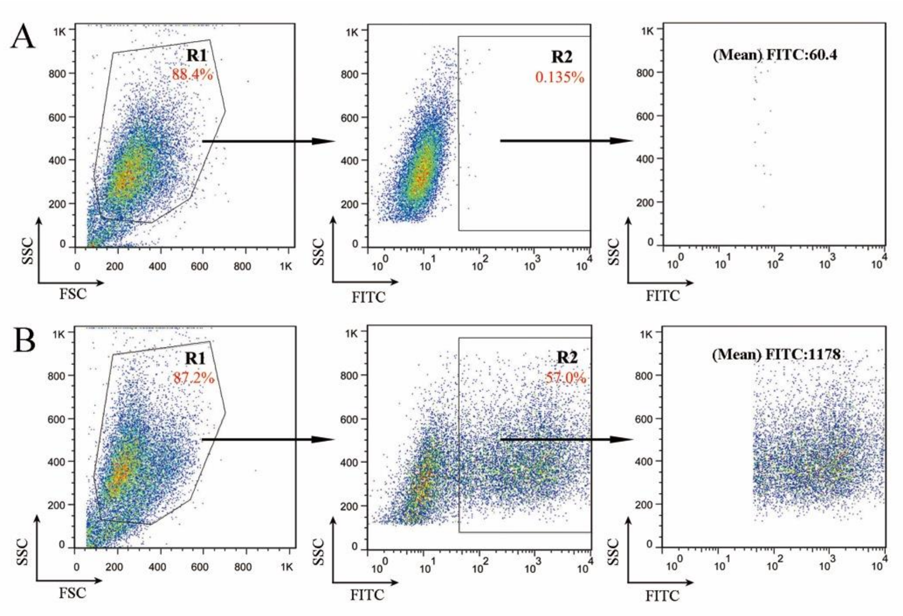

Figure S14 Flow cytometry on calculation of transfection efficiency and MFI of DC 2.4 cells treated without (A) or with (B) MP_{1000}-LPX NP 5. DC2.4 cells were captured via forward scatter (FSC) and side scatter (SSC). Live cells were gated as shown in Region 1 (R1), of which GFP positive cells were selected (R2). Transfection efficiency (% GFP^{+} cells) was auto displayed with R2. MFI of GFP was calculated based on the GFP positive cells population (R2) using FlowJo software.

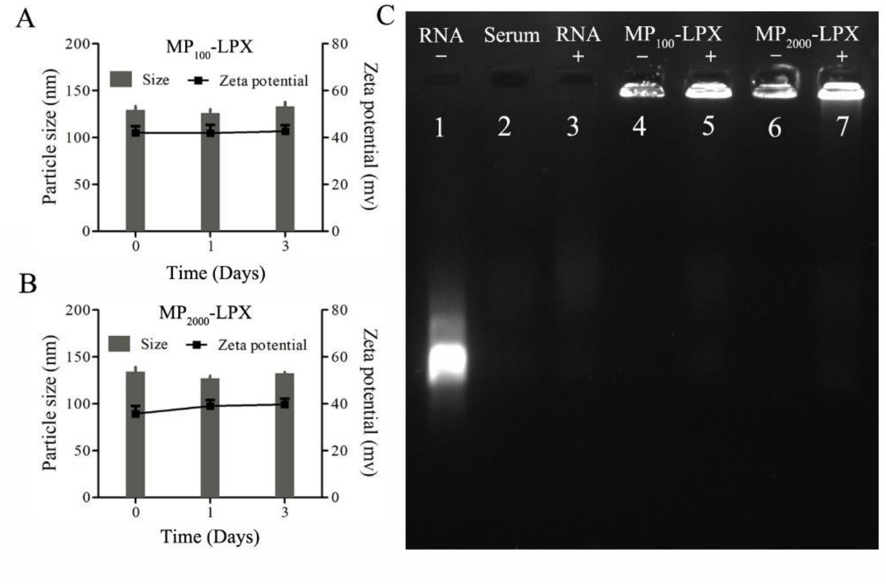

Figure S15 Stability of MP_{100}-LPX and MP_{2000}-LPX. Size and zeta potential measurements of MP_{100}-LPX (A) and MP_{2000}-LPX (B) at 0, 1, 3 days stored at 4°C. (C) Gel electrophoresis retardation assay to test serum stability. Free RNA (lane 1), Serum (lane 2), Serum + RNA (lane 3), MP_{100}-LPX (lane 4) and Serum + MP_{100}-LPX (lane 5),

and MP_{2000}-LPX (lane 6) and Serum + MP_{2000}-LPX (lane 7).

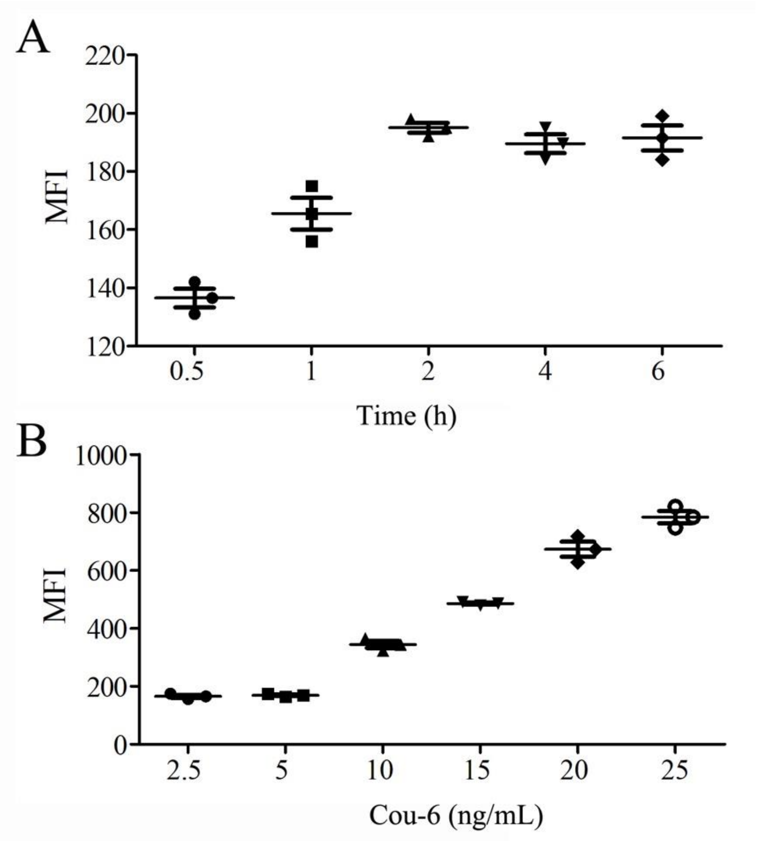

Figure S16 Cellular uptake of LPX on DC2.4 cells. (A) MFI of LPX (Cou-6) uptake by DC2.4 cells for different time. (B) MFI of LPX uptake by DC2.4 cells with different concentrations of Cou-6.
